# Creative ageing in a digital world: A protocol for a synthesis of arts-based innovations for culturally diverse older adults in Canada

**DOI:** 10.1371/journal.pone.0360724

**Published:** 2026-10-23

**Authors:** Kristina M. Kokorelias, Alisa Grigorovich, Kateryna Metersky, Areej Al-Hamad, Umair Rehman, Renato Barbosa dos Santos, Maurita T. Harris, Shelly-Anne Li, Stephanie Hatzifilalithis

**Affiliations:** 1 Department of Medicine, Healthy Ageing and Geriatrics Program, Sinai Health and University Health Network, Toronto, Ontario, Canada; 2 Department of Occupational Science and Occupational Therapy, Temerty Faculty of Medicine, University of Toronto, Toronto, Ontario, Canada; 3 Temerty Faculty of Medicine, Rehabilitation Sciences Institute, University of Toronto, Toronto, Ontario, Canada; 4 KITE, Toronto Rehabilitation Institute, University Health Network, Toronto, Ontario, Canada; 5 Brock University, St. Catharines, Ontario, Canada; 6 Daphne Cockwell School of Nursing, Toronto Metropolitan University, Toronto, Ontario, Canada; 7 Western University, London, Ontario, Canada; 8 Department of Physical Therapy, Temerty Faculty of Medicine, University of Toronto, Toronto, Ontario, Canada; 9 Wilfrid Laurier University, Brantford, Ontario, Canada; 10 University Health Network, Toronto, Ontario, Canada; 11 Women’s Age Lab, Women’s College Hospital, Toronto, Ontario, Canada; PLOS: Public Library of Science, UNITED KINGDOM OF GREAT BRITAIN AND NORTHERN IRELAND

## Abstract

The forthcoming scoping review will synthesize evidence on arts-based and digital engagement initiatives that support social inclusion, mental health, and cultural expression among older adults, with a particular focus on structurally-disadvantaged populations. Rapid digital transformation in the arts, including virtual and augmented reality, digital storytelling, and generative artificial intelligence, has expanded opportunities for participation and creative expression. However, access remains uneven, and there is limited consolidated evidence on effectiveness, equity considerations, and best practices for implementation across diverse contexts. Guided by Joanna Briggs Institute methodology and PRISMA-ScR reporting standards, the forthcoming review will systematically search peer-reviewed and grey literature across major health, social science, and interdisciplinary databases from inception to October 2026. An integrated knowledge translation approach will be embedded throughout the review, engaging older adults, caregivers, community arts practitioners, and cultural leaders as active partners in refining search strategies, interpreting findings, and shaping dissemination. Data will be extracted and synthesized using both quantitative mapping and qualitative content analysis to identify program models, theoretical frameworks, implementation strategies, outcomes, and equity considerations. Findings will generate a comprehensive evidence map of effective and emerging practices, highlight barriers such as digital exclusion and cultural underrepresentation, and identify ethical and legal considerations in digital arts engagement. The review will inform the development of culturally safe, accessible, and inclusive arts and digital programming, and will provide actionable guidance for researchers, practitioners, policymakers, and community organizations seeking to enhance well-being, social connection, and cultural continuity among older adults.

## Introduction

The arts have long played an important role in supporting social inclusion, cultural continuity, and personal expression across the life course [1]. Engagement in, and the creation of, art is also recognised as a core human right (e.g., Articles 24 and 27 of the Universal Declaration of Human Rights regarding the provision for access and equal opportunity to rest, leisure, and participation in the cultural life of their communities) [2]. For older adults, arts creation and participation can provide opportunities for connection, meaning, identity affirmation, and emotional well-being, particularly for populations who experience social or structural marginalization [3–5]. This includes disabled, 2SLGBTQIA+, racialized, immigrant, low-income, women, and Indigenous older adults, for whom mainstream programs may not always reflect cultural values, linguistic needs, or lived experience [3–5]. When opportunities for participation are not culturally relevant or accessible, older adults may face greater social isolation and exclusion. In contrast, arts-based engagement can create spaces for belonging, storytelling, intergenerational exchange, and the preservation of cultural heritage [1].

At the same time, the arts sector is undergoing rapid digital transformation. Emerging technologies such as virtual reality, augmented reality, digital storytelling platforms, online creative tools, and generative artificial intelligence are reshaping how arts are created, shared, and experienced. These technologies have expanded the possibilities for older adults to participate in creative activities, including those who face mobility limitations, geographic barriers, or care-related restrictions. Early examples suggest meaningful benefits [6]. Digital arts initiatives have been used to support reminiscence, stimulate cognitive engagement, and strengthen social connection, including projects that transform personal memories into visual or narrative artworks [7,8]. For some older adults, these approaches offer new ways to express identity and preserve life stories in formats that feel engaging, accessible, and personally relevant [9].

The COVID-19 pandemic accelerated this shift by prompting many arts organizations and community programs to move activities online or to develop hybrid and low-tech alternatives for remote participation [6]. While these adaptations helped maintain access for some older adults, they also exposed persistent inequities. Unequal access to devices, reliable internet, and digital skills continues to shape who can benefit from emerging technologies. In addition, many digital arts platforms still underrepresent culturally diverse content, which can limit their relevance for newcomer and racialized communities. Moreover, emerging evidence demonstrates that GenAI forms of art continue to amplify social exclusion and discrimination based on age, race, gender and disability [10,11]. For older adults who already face barriers to participation, the digital transition can either expand opportunities for inclusion or further reproduce exclusion if accessibility and cultural safety are not built into program design [1].

Indigenous-led digital arts initiatives offer important examples of how technology can be used in culturally grounded ways. These projects often emphasize relationality, community governance, language revitalization, and knowledge stewardship rather than simply digital access. In this context, digital storytelling and related creative practices are not only tools for expression but also forms of cultural continuity and self-determination [12,13]. However, Indigenous perspectives remain underrepresented in mainstream research on digital arts and aging, and there is limited synthesis of how such initiatives are being designed, implemented, and evaluated.

Although interest in arts-based and technology-enabled programming for older adults is growing, the evidence base remains fragmented. Existing studies vary widely in setting, population, intervention type, and outcome measurement, making it difficult to determine which approaches are most promising, for whom, and under what conditions. There is also limited clarity about the ethical, legal, and cultural considerations associated with emerging technologies such as generative AI, particularly in relation to authorship, ownership, representation, and cultural stewardship. These gaps are especially important in Canada, where older adults from diverse linguistic, cultural, and geographic contexts may experience distinct barriers and opportunities in relation to arts participation.

To address these gaps, this scoping review will map the current evidence on arts-based programs and their impacts on older adults. Specifically, it will identify the types of outcomes most commonly evaluated, as well as key gaps in the literature, with a focus on outcomes related to social inclusion, mental health, cultural expression, and overall well-being [14]. Particular attention will be given to the experiences of equity-deserving groups, including disabled, 2SLGBTQIA+, racialized, immigrant, low-income, women, and Indigenous older adults, across community, residential, and virtual care settings.. Using Joanna Briggs Institute methodology and reporting in accordance with PRISMA-ScR, the review will synthesize evidence on program types, settings, populations, theoretical approaches, outcomes, and implementation considerations. By identifying what is known, where the evidence is limited, and which approaches show promise, this review will inform future research, practice, and policy aimed at supporting socially inclusive, culturally relevant, and accessible arts engagement for older adults in Canada and beyond.

## Methods

This scoping review will be conducted in accordance with the methodological guidance of the Joanna Briggs Institute for scoping reviews [15,16] and reported following the PRISMA extension for Scoping Reviews (PRISMA-SCr) [17]. A scoping review approach is appropriate given the broad and heterogeneous nature of the literature on arts-based and digitally enabled engagement among older adults, and the need to map concepts, intervention characteristics, contexts, and knowledge gaps rather than evaluate intervention effectiveness alone.

Consistent with integrated knowledge translation principles, knowledge users will be engaged throughout the review process. An integrated knowledge translation panel composed of older adults, chosen family caregivers, community arts practitioners, and cultural or program leaders will provide input on search terminology, interpretation of findings, and dissemination planning. Particular attention will be given to perspectives relevant to racialized, newcomer, and Indigenous older adults to support the cultural relevance and applicability of the review.

Results of this study are anticipated in May 2027.

### Eligibility criteria

Studies will be eligible for inclusion if they report primary empirical research involving older adults, typically aged 55 years and older (as at least 50% of the population), participating in arts-based, digitally enabled, or hybrid creative programs intended to support social inclusion, mental health, cultural expression, participation, or well-being.

The review will exclusively focus on studies involving populations who may experience structural marginalization, including disabled, 2SLGBTQIA+, racialized, immigrant, low-income, women, and Indigenous older adults. Studies involving family members or chosen caregivers will be included when their participation is directly related to the intervention or program.

Eligible interventions will include, but will not be limited to, digital storytelling, virtual reality, augmented reality, generative artificial intelligence, online arts workshops, community-based arts initiatives, and other technology-enhanced creative programs. Studies conducted in community, residential care, long-term care, institutional, and virtual settings will be included.

We will include qualitative, quantitative, and mixed-methods primary studies, as well as program evaluations and case studies. Reviews, protocols, books, editorials, commentaries, opinion papers, and conference abstracts will be excluded, although their reference lists will be examined for potentially relevant primary studies.

### Information sources and search strategy

A comprehensive search strategy will be developed in collaboration with a health sciences information specialist. The search will be conducted across multiple bibliographic databases, including MEDLINE (Ovid), CINAHL (EBSCO), EMBASE (Ovid), and PsycINFO (Ovid), covering all records from database inception through October 2026. No language restrictions will be applied, and non-English studies will be translated as needed. In addition to peer-reviewed literature, we will hand-search targeted journals, conference proceedings, and grey literature sources, including OpenGrey, Grey Literature Report, GreyNet International, arXiv, bioRxiv, SSRN, and ProQuest Dissertations and Theses, to capture unpublished or non-indexed work relevant to older adults’ engagement with arts and digital technologies. The search will use keywords and subject headings related to three main concepts: older adults, arts-based engagement, and digital or technology-enhanced programs. The strategy will build on previous reviews and domain-specific terminology to ensure breadth and relevance. The final search strategy will be peer-reviewed by an information specialist using the Peer Review of Electronic Search Strategies (PRESS) checklist [18]. Additionally, the research team will leverage professional and community networks to identify further relevant studies, ensuring comprehensive coverage of published and grey literature.

### Study selection

All references identified through the search will be imported into Covidence and duplicates removed. A pilot test of the level 1 screening form, based on the inclusion and exclusion criteria, will be conducted on a random sample of 25% of records. Eligibility criteria will be refined if necessary to improve clarity and consistency, particularly if inter-rater agreement is below 70%. Screening will be conducted in two stages, each independently by two reviewers. In level 1, titles and abstracts will be assessed for potential relevance. At level 2, full texts of articles that pass the initial screen will be reviewed to determine final inclusion. A pilot test of the level 2 screening form will also be conducted on approximately 25% of full-text articles, with inter-rater reliability calculated. Discrepancies at either stage will be resolved through discussion, and a third reviewer will adjudicate unresolved conflicts. Excluded studies will be documented, along with the reasons for exclusion.

We anticipate study selection to be completed by January 2027.

### Critical appraisal

To assess the quality, accuracy, and rigor of current work in the field, we will systematically evaluate the methodological approaches of included studies using established tools. The Joanna Briggs Institute critical appraisal checklists will be applied to quantitative, qualitative, and mixed-methods studies, as these tools provide structured, validated criteria for assessing study design, sampling, data collection, analysis, and reporting transparency [19]. For qualitative studies, the CASP (Critical Appraisal Skills Programme) checklist will additionally be used to evaluate credibility, relevance, and reflexivity in study design. While no studies will be excluded based on quality, the appraisal will document strengths, such as robust or culturally sensitive methods, and identify gaps, including limited sample diversity, inconsistent outcome measures, or underrepresentation of marginalized older adults [20].

### Data extraction

Data from included studies will be extracted using a standardized charting form developed iteratively by the research team and pilot-tested prior to full extraction. The form will capture descriptive and analytical information relevant to the objectives of the review, including bibliographic characteristics, country of study, study aims, design, setting, participant characteristics, intervention or program features, theoretical or conceptual frameworks, and reported outcomes related to social inclusion, cultural expression, participation, mental health, or well-being.

To support the equity-focused aims of this review, the extraction framework will include additional fields designed to capture how studies characterize participant populations and whether findings are reported for specific groups. In particular, studies will be coded according to whether they explicitly include disabled, 2SLGBTQIA+, racialized, immigrant, low-income, women, and Indigenous older adults. Where studies include heterogeneous samples, we will document whether subgroup-specific findings are presented. When subgroup-specific analyses are not reported despite the inclusion of these populations, this will also be recorded as an important characteristic of the evidence base.

The extraction framework will also include variables related to cultural safety, language accessibility, and digital access. Cultural safety will be coded by documenting whether interventions are described as culturally tailored, culturally adapted, culturally grounded, or community-led, and whether cultural knowledge holders, Elders, or community partners were involved in intervention design, delivery, or governance. We will also extract information on whether studies explicitly address cultural relevance, cultural continuity, cultural ownership, or Indigenous community protocols.

Language-related variables will capture the language or languages used in intervention delivery, as well as the use of translated materials, interpretation, subtitles, bilingual facilitation, or other language-access supports. Where language is described as influencing participation, it will be recorded as either a facilitator or barrier.

To assess digital inclusion, we will extract information related to participants’ access to devices, internet connectivity, and other technological infrastructure, as well as reported levels of digital literacy or familiarity with digital tools. We will also document whether studies describe supports such as technical assistance, training, coaching, simplified interfaces, or low-technology alternatives. Where digital access is discussed as influencing participation or engagement, this will be coded as either a facilitator or barrier.

The charting form may be refined during the review process to ensure that emerging concepts relevant to culturally safe and digitally inclusive arts engagement are captured consistently across studies. The abstraction form will be pilot tested and refined by at least two members of the research team to enhance clarity and consistency. To ensure transparency and reliability, inter-rater agreement will be assessed by calculating Cohen’s κ and percentage agreement, with discrepancies resolved through discussion or consultation with a third reviewer as needed [21].

Additional categories may be added through consultation with the research team and the integrated knowledge translation (iKT) panel to ensure relevance to older adults, marginalized populations, and digital or arts-based interventions.

Data extraction anticipated to be completed by March 2027.

### Data analysis

Data extracted from included studies will be analyzed using a convergent mixed-methods approach [22] that combines descriptive numerical mapping with qualitative content analysis [23], consistent with the objectives of a scoping review. Quantitative and qualitative findings will first be analyzed separately and then integrated during interpretation to provide a comprehensive understanding of how emerging arts-based and digital approaches are being used with older adults across diverse settings and populations.

Quantitative data will be summarized using numerical counts and descriptive statistics. These analyses will characterize the distribution of studies by publication year, country, study design, care setting, participant characteristics, and intervention type. We will also map the frequency with which specific technologies are reported, including virtual reality, augmented reality, digital storytelling, online arts platforms, and generative artificial intelligence. Additional descriptive analyses will examine the extent to which studies report cultural tailoring, multilingual delivery, digital-access supports, or explicit attention to cultural safety. Where feasible, cross-tabulations will be used to explore patterns across study characteristics, such as the relationship between intervention type and setting, or between participant subgroup and reported outcomes.

Qualitative data will be analyzed using directed content analysis [24]. An initial coding framework will be developed deductively from the review objectives and extraction domains, including participant engagement, intervention design, cultural relevance, language accessibility, digital inclusion, and reported outcomes. During analysis, inductive codes will also be added to capture unanticipated concepts that emerge from the literature. Codes will be grouped into higher-order categories and themes to identify patterns in how arts-based and digitally enabled interventions are conceptualized, implemented, and experienced.

The qualitative synthesis will focus on several interrelated analytic questions. First, we will examine how older adults were engaged in arts and digital programs, including whether participants were positioned primarily as recipients, collaborators, co-designers, storytellers, or creators. Particular attention will be paid to strategies used to facilitate participation among disabled, 2SLGBTQIA+, racialized, immigrant, low-income, women, and Indigenous older adults, including community partnerships, culturally grounded facilitation, peer support, and intergenerational engagement.

Second, we will analyze the theoretical, conceptual, or practice-based frameworks guiding program design and evaluation. This may include frameworks related to social inclusion, cultural safety, healthy aging, participatory arts, digital inclusion, community engagement, or Indigenous knowledge systems. Mapping these frameworks will help identify the assumptions and principles that shape intervention development and interpretation of outcomes.

Third, we will examine how studies combine arts-based approaches with emerging technologies to support social inclusion, mental health, cultural expression, and well-being. This analysis will consider intervention format, mode of delivery, the role of technology within creative processes, and whether technologies are used primarily to expand access, enhance immersion, support storytelling, facilitate reminiscence, or promote cultural preservation.

A specific analytic focus will be placed on equity-related subgroup analysis. Where sufficient data are available, subgroup analyses will be conducted to explore how interventions and outcomes differ across disabled, 2SLGBTQIA+, racialized, immigrant, low-income, women, and Indigenous older adult participants. We will examine whether these studies report distinct patterns in cultural tailoring, language accessibility, digital access, community leadership, barriers to engagement, or reported psychosocial and cultural outcomes. Where subgroup-specific findings are not explicitly reported despite inclusion of these populations, this absence will be documented as an evidence gap. In addition, subgroup analyses will be undertaken where possible according to age, gender, ethnicity, cultural background, language, socioeconomic status, and care setting. These analyses are intended not to test statistical differences, but rather to map how participant characteristics shape the contexts, accessibility, and reported impacts of arts and digital engagement initiatives.

Findings related to cultural safety, language, and digital access will also be synthesized across studies. This will allow us to identify recurring barriers and facilitators, such as language concordance, translation supports, internet connectivity, access to devices, digital literacy, or the presence of culturally meaningful content. Particular attention will be paid to whether these factors are treated as peripheral implementation issues or as central determinants of participation and inclusion. The final synthesis will integrate quantitative and qualitative findings through evidence tables, evidence maps, and narrative summaries. These outputs will be used to identify areas of concentration in the literature, describe emerging models of practice, highlight barriers and implementation considerations, and identify important gaps requiring future research. The synthesis will therefore provide both a descriptive map of the field and a more interpretive understanding of how emerging technologies are being used to support equitable and culturally meaningful arts engagement among older adults.

### Integrated knowledge translation

This scoping review will be guided by an integrated knowledge translation (iKT) approach to ensure that the review questions, interpretation of findings, and dissemination activities remain relevant to the needs and priorities of intended knowledge users [25]. Consistent with participatory approaches to evidence synthesis, knowledge users will be engaged throughout the project rather than only at the dissemination stage. An iKT panel will be established in collaboration with the research team and will include older adults, chosen family caregivers, community arts practitioners, cultural leaders, and program administrators with experience supporting diverse older adult populations. Effort will be made to include perspectives relevant to disabled, 2SLGBTQIA+, racialized, immigrant, low-income, women, and Indigenous older adults. Panel members will be recruited through existing academic, community, and cultural networks.

The iKT panel will contribute at multiple stages of the review. During protocol refinement, panel members will provide input on terminology, concepts, and sources that may strengthen the relevance and comprehensiveness of the search strategy, particularly for culturally specific and community-based programming that may be underrepresented in conventional academic databases. During data interpretation, panel members will help contextualize emerging findings, identify issues of practical importance, and interpret results through the lens of lived experience, cultural relevance, accessibility, and implementation feasibility. Their input will also inform the development of dissemination outputs to ensure that findings are communicated in ways that are meaningful and usable across different sectors. This collaborative approach is intended to strengthen the interpretive depth, cultural responsiveness, and practical relevance of the review, while supporting reciprocal exchange between academic and community-based forms of knowledge.

At the completion of the review, findings will be disseminated through an end-of-grant knowledge translation strategy designed to reach academic, policy, community, and practice audiences. Academic dissemination will include publication in a peer-reviewed journal and presentations at national and international conferences focused on aging, arts, digital health, and community engagement. These outputs will emphasize the scope of the evidence base, methodological gaps, and implications for future intervention research. To support practice and community uptake, the research team will develop plain-language summaries, evidence tables, and visual evidence maps describing intervention types, participant groups, settings, and key findings related to social inclusion, cultural expression, mental health, and accessibility. These materials will be shared with community organizations, arts practitioners, and older adult networks. In collaboration with the iKT panel, community-facing dissemination activities will also include webinars, stakeholder dialogues, and targeted workshops with community and cultural organizations. These activities will focus on translating findings into practical implications for culturally relevant program design, digital inclusion, and equitable access to arts participation for older adults.

## Discussion

This scoping review will provide a comprehensive synthesis of the existing literature on arts-based strategies for older adults and their impacts, with particular attention to disabled, 2SLGBTQIA+, racialized, immigrant, low-income, women, and Indigenous older adults. Guided by the methodological framework of the Joanna Briggs Institute and reported in accordance with PRISMA-ScR, the review is intended to map the breadth, range, and characteristics of current evidence rather than evaluate intervention effectiveness. By focusing on emerging technologies in arts engagement, the review responds to growing interest in how digital innovation may support social inclusion, mental health, cultural expression, and participation among older adults across community, residential, long-term care, and virtual settings.

We anticipate that the review will identify a rapidly expanding but methodologically heterogeneous body of literature. Emerging technologies such as virtual reality, augmented reality, digital storytelling, online creative platforms, and generative artificial intelligence are expected to appear across a range of arts-based initiatives designed to support engagement, reminiscence, cultural preservation, and social connection. The literature is likely to show that digital and hybrid arts programs can offer meaningful opportunities for older adults to participate in creative expression, particularly for those who are homebound, geographically isolated, or living in care settings. At the same time, we anticipate that the evidence will reveal important inequities in access and implementation. Persistent barriers related to digital literacy, access to devices and internet connectivity, language accessibility, and culturally relevant content are expected to emerge as recurring themes. These barriers may be particularly salient for racialized, newcomer, and Indigenous older adults, whose participation in arts and technology programs may be shaped by broader structural inequities. We also anticipate identifying promising examples of culturally grounded and community-led approaches. Indigenous-led digital storytelling and cultural media initiatives may illustrate how emerging technologies can support language revitalization, knowledge stewardship, and cultural continuity when embedded within community governance and relational ways of knowing. However, subgroup-specific reporting is expected to remain limited, and many studies may include diverse participant groups without disaggregating findings by ethnicity, migration background, language, or cultural identity.

A major strength of this review is its explicit focus on equity, cultural relevance, and digital inclusion. By examining not only intervention types and outcomes but also how cultural safety, language accessibility, and digital access are addressed, the review will extend beyond conventional mapping of arts-based interventions and offer a more nuanced understanding of participation and inclusion. A second strength is the deliberate emphasis on racialized, newcomer, and Indigenous older adults. Although these populations are often underrepresented or aggregated within broader aging research, this review will explicitly examine how they are represented in the literature and whether findings are reported in ways that allow meaningful subgroup interpretation. The use of an integrated knowledge translation approach is also a key strength. Involving older adults, caregivers, community arts practitioners, and cultural leaders throughout the review process will enhance the practical relevance and interpretive depth of the findings. This is particularly important in a field where community knowledge, cultural context, and lived experience are central to understanding what constitutes meaningful engagement. Finally, the inclusion of peer-reviewed and grey literature will improve the comprehensiveness of the review by capturing community-based, practice-informed, and emerging work that may not yet be fully represented in traditional academic publication channels.

Several limitations should be acknowledged. First, as a scoping review, this study is intended to map evidence rather than determine effectiveness or establish causal relationships. The review will therefore not provide definitive conclusions regarding the comparative effectiveness of specific technologies or intervention models. Second, the quality and depth of synthesis will depend on the characteristics of the available literature. Existing studies may vary considerably in methodological rigor, outcome measurement, reporting quality, and conceptual clarity. Inconsistent reporting of participant demographics may limit subgroup analysis and constrain interpretation of how racialized, newcomer, or Indigenous older adults specifically experience these interventions. Third, although no language restrictions will be applied, the review may still under capture culturally embedded community initiatives that are unpublished, locally disseminated, or not easily retrievable through academic and grey literature databases. This limitation may be especially relevant for Indigenous and community-led arts initiatives whose outputs may not conform to conventional research dissemination formats. Finally, the rapidly evolving nature of emerging technologies means that the evidence base may change quickly. New platforms, tools, and practices may emerge during or shortly after the review process, which is an inherent challenge in fields shaped by ongoing technological innovation.

## Conclusion

This scoping review will provide an important synthesis of current knowledge on how emerging technologies are being integrated into arts-based engagement with older adults. By mapping intervention characteristics, participant populations, equity-related factors, and reported outcomes, the review will help clarify how digital innovation may support social inclusion, cultural continuity, mental health, and creative participation in later life. The anticipated findings are expected to highlight both the promise and the unevenness of current practice. While emerging technologies may create new opportunities for expression, connection, and cultural preservation, the review is also likely to underscore the continued importance of cultural safety, language accessibility, community leadership, and digital equity. Ultimately, this review will establish an evidence-informed foundation for future research, program development, and policy planning. In doing so, it will contribute to ongoing efforts to ensure that the digital transformation of arts and culture supports inclusive, culturally meaningful, and accessible participation for older adults in Canada and beyond.

## Supporting information

S1 File PRISMA ScR Fillable Checklist AUG2026. (PDF)

S2 File 20260619 Ovid MEDLINE. (DOCX)
