# Supplementary material for: Creative ageing in a digital world: A protocol for a synthesis of arts-based innovations for culturally diverse older adults in Canada
Source: PLoS One. 2026 Oct 23;21(10):e0360724. doi: 10.1371/journal.pone.0360724 (PMC13693869; doi:10.1371/journal.pone.0360724)
Supplement: Supplementary file 2 [file pone.0360724.s002.docx]

Ovid MEDLINE(R) ALL <1946 to June 18, 2026>

1	exp Art/ or exp Caricatures as Topic/ or Music/ or Writing/ or Drama/ or Poetry as Topic/	80273

2	(art or stage* or "design" or "designed").ti.	420820

3	("visual art" or arts or artistic* or "art work" or artworks or (work* adj1 art*) or caricature* or cartoon* or engrav* or illustrat* or paint* or "pictorial work*" or portrait* or sculpt*).ti.	32256

4	(music* or song* or "vocal melod*" or sing or singing or choir* or choral* or "classical music" or jazz or "hip hop" or (rock adj1 roll)).ti.	27912

5	(write* or wrote* or drama* or theatre*).ti.	13619

6	(pottery* or craft* or draw* or sketch* or storytell* or "story tell*").ti.	16236

7	(dance or dancing or ballet or (tap adj1 danc*)).ti.	5270

8	(art* adj1 (event* or communit* or program* or engag* or creat* or perform* or show*)).ti,ab,kf,kw.	31017

9	or/1-8	598101

10	Aged/ or "Aged, 80 and Over"/ or Frail Elderly/	3895763

11	(aged* or senior* or elder* or "oldest old" or "old* people" or "old* person" or "old* adult" or geriatric* or grandparent* or retire* or pensioner* or centenarian* or supercentenarian* or semisupercentenarian* or nonagenarian* or octogenarian*).ti,ab,kf,kw.	1393005

12	10 or 11	4720278

13	Generative Artificial Intelligence/ or Virtual Reality/ or Augmented Reality/ or Digital Technology/ or Mobile Applications/ or exp Computers, Handheld/ or Online Systems/	55539

14	((emerg* adj3 technolog*) or "digital technolog*").ti,ab,kf,kw.	36420

15	((virtual* or augment* or mixed or extend*) adj2 realit*).ti,ab,kf,kw.	35165

16	("generative ai" or "gen* ai" or genai).ti,ab,kf,kw.	4362

17	(chatbot or "chat bot" or chatgpt or "chat gpt").ti,ab,kf,kw.	12439

18	VR.ti.	1633

19	("mobile app" or "mobile application*" or "electronic app" or "electronic application*" or "phone app" or "phone application*" or "smartphone app" or "smartphone application*" or "digital app" or "digital application*").ti,ab,kf,kw.	25512

20	(smartphone* or "smart phone" or tablet* or "mobile device").ti,ab,kf,kw.	110484

21	(online or digital* or virtual* or remote*).ti,ab,kf,kw.	907653

22	or/13-21	1062614

23	exp Persons with Disabilities/ or exp "Sexual and Gender Minorities"/ or exp Minority Groups/ or exp "Emigrants and Immigrants"/ or Poverty/ or Women/ or Indigenous Peoples/ or exp Indigenous Canadians/	207231

24	(disabilit* or disabled* or handicap*).ti,ab,kf,kw.	352028

25	pwd.ti,ab.	2930

26	(amputee* or deaf or (hard* adj1 hear*) or (hear* adj1 impair*) or blind or (visual* adj1 impair*)).ti,ab,kf,kw.	306878

27	((minor* or divers* or dissident*) adj2 (sexual* or gender*)).ti,ab,kf,kw.	17624

28	(glbt or lgbt ot lgbtq or lgbtqia).ti,ab.	683

29	(lesbian or queer or gay or bisexual or asexual or "non heterosexual" or homosexual*).ti,ab,kf,kw.	48172

30	("non binary" or nonbinary or "gender non conform*" or intersex or transgender* or transexual* or "two spirit" or transmasculine or transfeminine).ti,ab,kf,kw.	22653

31	(minor* adj2 (group* or gender* or sexual* or racial* or ethnic*)).ti,ab,kf,kw.	45510

32	(bipoc or black or indigenous).ti,ab,kf,kw.	269204

33	(emigra?t* or immigra?t* or foreigner* or refugee* or asylum* or alien).ti,ab,kf,kw.	82179

34	(poverty or "low income" or "low* socioeconomic*").ti,ab,kf,kw.	116588

35	(women* or woman* or female*).ti,ab,kf,kw.	2903322

36	or/23-35	3933215

37	9 and 12 and 22 and 36	857

38	9 and 12 and 22	3371

39	limit 38 to yr="2016 -Current"	2516
